# Regulatory Mechanisms of Transcription Factors in Plant Morphology and Function

**DOI:** 10.3390/ijms24087039

**Published:** 2023-04-11

**Authors:** Tomotsugu Koyama

**Affiliations:** Bioorganic Research Institute, Suntory Foundation for Life Sciences, Seikacho, Kyoto 619-0284, Japan; koyama@sunbor.or.jp

Plants develop organs such as flowers and leaves with different morphologies. For plant survival, the morphogenesis of these organs is genetically programmed and flexibly changed in response to external stimuli [1]. Morphogenesis is largely achieved by the temporal and spatial activation or repression of the transcription of genes used for regulators and enzymes [2]. Therefore, the importance of transcription factors (TFs) in the morphogenesis is evident. This Special Issue underscores the regulatory mechanisms of TFs in plant morphology and function. This Special Issue also helps to identify important TFs for morphogenesis and clarify their genetic regulatory cascades for morphogenesis. Other contributions in this Special Issue offer insights into the integration of TFs and plant hormone signals for morphogenesis.

TFs tightly control plant morphogenesis and flexibly alter it in response to external stimuli. Pautot et al. demonstrated the role of SINGLE LEAFLET (SGL1), a member of the LEAFY TFs, in the morphogenesis of the leaves and petals of *Medicago truncatula* [3]. They compared the roles of SGL1 and SHOOT MERISTEMLESS, a KNOX-like TF, in the formation of the compound leaves of *M. truncatula*. Leaf morphological changes occur in response to environmental signals. Legume leaf movement results from the deformation of pulvinar cells. Nakata and Takahara reviewed pulvinar deformation and development, the latter of which is regulated by the complex actions of TFs [4]. *Rorippa aquatica* changes its leaf morphology to narrow blades in response to submergence. Sakamoto et al. indicated that submergence downregulates signals for ANGUSTIFOLIA3/GROWTH-REGULATING FACTOR TFs, which regulate cell proliferation and expansion during leaf morphogenesis [5]. Vegetative shoot meristems develop leaves, whereas inflorescence meristems develop flowers and other reproductive organs. Gonzalez et al. reported global changes in gene expression during the vegetative-to-inflorescence meristem transition of the common bean (*Phaseolus vulgaris*) [6]. They reported that APETALA1, LEAFY, and CONSTANS-LIKE5 TFs are markers that distinguish between vegetative and inflorescence meristems.

TFs also integrate plant hormone signaling into plant morphological regulation. Shi et al. reviewed recent advances in the functions of the BES1/BZR1 family TFs in brassinosteroid (BR)-dependent and -independent signaling for plant development [7]. Viola et al. comprehensively reviewed the roles and mechanisms of class I TCP TFs in the integration of hormone signals during development, defense, and abiotic stress responses [8]. AGAMOUS-like 15 (AGL15) is a member of the MADS domain of TFs and plays an important role in the regeneration of somatic embryogenesis, which is often utilized in biotechnological applications. Joshi et al. analyzed the target genes of AGL15 using next-generation sequencing and identified the BR signals downstream of AGL15 [9]. Ethylene affects young seedling morphology and Longo et al. investigated potential inhibitors of histone acetyltransferases, which are involved in the epigenetic positive control of gene regulation in ethylene signaling and hypocotyl elongation in *Arabidopsis thaliana* [10]. 

TFs regulate programmed cell death and senescence, both of which are important for plant development. Senescence is associated with color changes in organs and is involved in the translocation of nutrients from old tissues to newly growing tissues and storage organs. Andrade Galan et al. reported the involvement of HD-ZIP III TF REVOLUTA in leaf morphogenesis and senescence via the interaction with a member of the TIFY family of proteins [11]. The inhibition of senescence increases the commercial value of ornamental plants. Takahashi et al. identified a NAC domain TF, EPHEMERAL1, in Japanese gentians whose transcript levels increased with age [12]. They generated a knockout mutant of *EPHEMERAL1* in Japanese gentians and confirmed improved flower longevity. Pollen development is important for reproduction and is mediated by programmed cell death in the tapetum of anthers. Guo et al. characterized an MYB TF that regulates tapetal cell death and is involved in pollen development [13].

Finally, TFs regulate plant responses to abiotic stress. Shen et al. explored the functions of SNF-related protein kinase2 and type A protein phosphatase 2C families in the abscisic acid signaling pathway and drought stress response in soybean plants [14].

In this research topic, impressive research and review papers have led us to investigate the roles of TFs in various aspects of plant morphogenesis and function. Fascinating progress has been made, but scientific questions remain in this research area, such as how TFs regulate heterogeneous cell populations for plant morphogenesis and how TFs coordinate individual biochemical and mechanical processes [15,16]. Future studies will uncover the regulatory mechanisms of TFs in plant morphogenesis and allow the development of genome-edited plants that are feasible for biotechnological applications.
